# Cavefishes in Chronobiological Research: A Narrative Review

**DOI:** 10.3390/clockssleep5010007

**Published:** 2023-02-10

**Authors:** Vera V. Pavlova, Viacheslav V. Krylov

**Affiliations:** 1 Papanin Institute for Biology of Inland Waters, Russian Academy of Sciences, 152742 Borok, Russia; 2 Scientific and Technological Center of Unique Instrumentation, Russian Academy of Sciences, 117342 Moscow, Russia

**Keywords:** *Astyanax mexicanus*, circadian clock, behavior, rhythm, caves, light–dark cycle

## Abstract

Cavefish are vertebrates living in extreme subterranean environments with no light, temperature changes, and limited food. Circadian rhythms in these fish are suppressed in natural habitats. However, they can be found in artificial light–dark cycles and other zeitgebers. The molecular circadian clock has its peculiarities in cavefish. In *Astyanax mexicanus*, the core clock mechanism is tonically repressed in the caves due to the overactivation of the light input pathway. A lack of functional light input pathway but rather the entrainment of circadian genes’ expression by scheduled feeding were revealed in more ancient *Phreatichthys andruzzii*. Different evolutionarily determined irregularities in the functioning of molecular circadian oscillators can be expected in other cavefish. The unique property of some species is the existence of surface and cave forms. Along with the ease of maintenance and breeding, it made cavefish a promising model for chronobiological studies. At the same time, a divergence of the circadian system between cavefish populations requires the strain of origin to be indicated in further research.

## 1. Introduction

Most of the model organisms used in chronobiological studies (such as fruit flies, zebrafish, and mice) are species that live in a daily changing environment. The circadian rhythms of their behavior and physiological and biochemical processes strictly follow the internal oscillators, which, in turn, is synchronized with the external light–dark cycle. However, animals from arrhythmic environments, such as cavefish, may be more suitable for some specific scientific purposes.

According to information from the website https://cavefishes.org.uk/ (accessed on 8 February 2023) which aggregates information about cavefish from 1436 to the present, “there are currently 296 species of cave and groundwater fishes, 53 species of interstitial fishes, 49 species with troglomorphic features from non-subterranean habitats” [1]. The most abundant cavefish are representatives of the Cypriniformes and Siluriformes orders: 139 and 89 species, respectively. Cavefish dwell in every continent except Antarctica and generally are restricted to tropical and subtropical regions. Caves in South America and Asia are the most populated, and only two cave species live in Europe [2].

There are two opposing hypotheses regarding the origin of hypogean fauna. The climate relict hypothesis says that caves served as refuges for epigean animals from inhospitable conditions. Over time, hypogean dwellers achieved troglomorphic traits, and their epigean ancestors went extinct [3]. *Phreatichthys andruzii*, *Garra barreimiae* [4], and *Indoreonectes evezardi* [5] are species considered to evolve this way. According to the adaptive shift hypothesis, cave species evolved from surface ancestors during the occupying of new ecological niches to reduce competition [3]. *Astyanax mexicanus* and *Poecilia mexicana* [4] are examples of this way, as they have recent epigean morphotypes.

Caves are specific habitats without usual surface environment rhythms. Troglobionts experience a lack of light–dark cycles. There are no valuable changes in temperature and water quality in cave water bodies [6]. Some rhythmicity in subterranean habitats may be provided by troglophiles. For example, the activity of bat populations in the caves could be a possible zeitgeber for troglobites. These animals show increased activity when leaving and returning to caves around sunrise and sunset. A flock of bats forms noise and vibrations. In addition, their rhythmic defecation during flights in caves may provide a feeding cue for cavefish. However, *per1* expression analysis of *A. mexicanus* field samples from Chica Cave, which has a highly rhythmic bat population, showed no rhythms related to bat activity [7]. The cave environment led to some adaptations arising which are convergent in many troglobites. The most pronounced alterations in cavefish are the loss of vision and pigmentation. These adaptations are achieved by different mutations in isolated populations [2,8]. The feature of cave fishes is the intensification of non-visual sensing (primarily mechanoreception). It is critical for orientation and food detection in the absence of eyes.

Circadian rhythms seem unnecessary in unchanging cave habitats. However, depending on the rate of evolutionary transformations, one can expect different features of the circadian system associated with a reduction of biological rhythms in different troglobites. The objective of this review was to analyze the available information about circadian rhythms in cavefish species. We collected information about the rhythms in the behavior of cavefish, analyzed data on the features of the molecular clock in these fish, and considered evolutionary aspects of the degree of change in circadian rhythms in different cave species in comparison with surface forms.

## 2. Results

### 2.1. Rhythms in Cavefish Behavior

Despite the lack of light cues in caves and the reduction of the visual analyzer, cavefish can exhibit circadian rhythms in laboratories. Periodicity in locomotor activity is well studied in several troglobite fish species (Table 1).

Thus, cavefish *Indoreonectes* (*Nemacheilus*) *evezardi* exhibit bimodal circadian rhythms of locomotor activity in the 12:12 light–dark cycle [14]. Cosinor rhythmometry revealed significant 12 h and 24 h activity rhythms. According to spectrum analysis, prominent periods of the most studied fish were 12 h or 24 h; one fish exhibited a 26.18 h period. The free-running rhythms showed increased variability among seven studied fish in total darkness. Cosinor analysis revealed that two fish retained both 24 h and 12 h rhythms, two other fish kept only the 24 h rhythm, and three fish showed no significant rhythm [14].

Several papers reported that feeding schedule could serve as zeitgeber in *I. evezardi*. A study by Biswas and Ramteke [15] carried out in constant darkness showed that the rhythmicity of vertical swimming activity depended on feeding patterns. Fish that were fed once a day at 18:00 demonstrated a 24 h period with an acrophase near 18 o’clock. Pradhan and co-authors [20] showed that the feeding regime could modulate circadian activity rhythms in *I. evezardi*. A peak in phototactic activity occurred at different times depending on the feeding. Feeding rhythmicity may be found in caves due to the bat populations living there, such as in Kotumsar cave, the only habitat of *I. evezardi* [15]. As written above, bat populations may provide rhythmic defecation timed to bursts of activity around dawn and dusk as they enter and leave the cave [7]. However, this assumption needs further research.

Cave individuals of *I. evezardi* also demonstrate behavioral rhythm in burying activity that reaches its peak at subjective dawn [21]. The air-gulping was rhythmic in this species for 9 out of 14 months of observation under constant darkness [22].

Circadian rhythms were also reported in *I. evezardi* physiological processes. A 24 h rhythm was revealed in the levels of glycogen and lactate in muscle and the acetylcholinesterase activity in gill and brain tissues under the 12:12 light–dark cycle [23]. Pradhan and Biswas [24] showed that the state of chromatophores gradually changes in this species during the day. The degree of pigmentation reaches its maximum in the latter half of the light phase.

**Figure 1:**
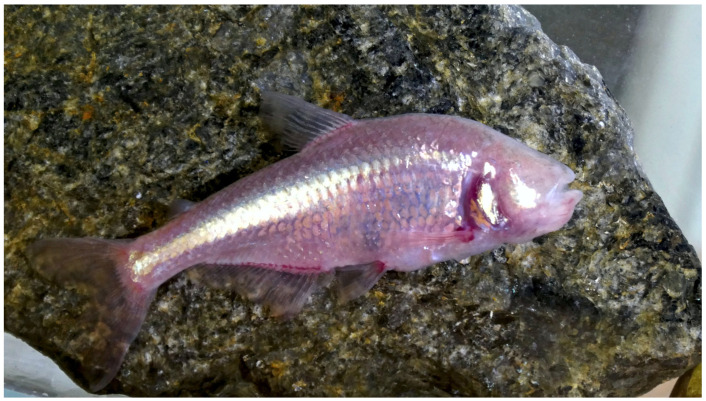
Blind Mexican tetra *Astyanax mexicanus*.

Blind Mexican tetra *A. mexicanus* (Figure 1) is the most popular model species in a wide range of research fields due to the ease of maintenance and breeding and the presence of two sharply different morphotypes: surface and cave [25]. The cave form of *A. mexicanus* entrained to a 12:12 light–dark cycle showed significant rhythm in locomotor activity with periods 23.95 ± 0.82 h and 25.6 ± 1.30 h in fish from Pachón and Chica caves, respectively [7]. Erckens and Martin [16] showed that locomotor activity could be entrained by different external light–dark regimes. Despite a high level of background noise, activity peaks with periods close to 24 h, 18 h, and 10 h were demonstrated in *A. mexicanus* after entrainment by 12:12, 9:9, and 3:7 light–dark cycles, respectively [16].

Fish maintained under a 12:12 light–dark cycle from fertilization to the age of 6–12 months also exhibited significant rhythmicity in both light–dark conditions and total darkness. At the same time, the individuals reared in the darkness did not possess any rhythmicity [26]. Rhythms were also detected in spatial preferences. More fish prefer the bottom of a tank at night than during the day [16,26].

Duboué and Borowsky [17] studied three balitorid species from Thailand caves. All of them demonstrated circadian rhythms in locomotor activity in darkness. These rhythm periods were 23.19 ± 3.11 h for *Schistura jaruthanini*; 24.06 ± 0.19 h for *S. spiesi*; and 24.92 ± 1.03 h for *Nemacheilus troglocataractus*. Moreover, *S. oedipus* demonstrated an infradian rhythm with a 38.50 ± 1.25 h period.

Brazilian blind cave catfishes *Pimelodella kronei*, *Imparfinis* sp. (=*Rhamdiopsis krugi*) [11,27], *Trichomycterus* sp. [10], and *Taunayia* sp. (=*Rhamdiopsis* sp.) [12,27] were also studied. Among these species, some individuals exhibited circadian rhythm in constant darkness, while others did not. Ultradian and infradian rhythms were often registered in these fish. Brazilian cavefishes are an example of the gradual evolution of troglobiont traits: from *Pimelodella spelea* with reduced eyes to *Stygichthys typhlops* with total loss of pigmentation and visual structures. The degree of specialization to subterranean life determines the robustness of circadian rhythmicity: the more specialized species, the less circadian rhythmicity was exhibited [9,27].

A Somalian cavefish *Phreatichthys andruzzii* evolved from surface-dwelling ancestors at least a million years ago [28]. During this time, it has acquired stable troglobiont features. Adult fishes lack eyes, pigments, and scales [29]. A comprehensive investigation by Cavallari with co-authors [18] revealed that this fish demonstrates no circadian rhythms entrained by the light–dark cycle. In contrast to the above described rhythms in *I. evezardi* and *A. mexicanus* entrained by the light–dark cycle, its locomotor activity is arrhythmic. This is perhaps due to the more ancient origin of *P. andruzzii*. However, this species exhibits a significant food-entrainable rhythm [18].

### 2.2. Molecular Circadian Oscillations in Cavefish

It is obvious that such differences in circadian rhythms are a reflection of the peculiarities of endogenous molecular oscillators in cavefish. The existence of surface and cave forms within the same fish species helps to understand the features of the molecular clock in an arrhythmic habitat.

Beale et al. [7] studied the circadian clock function in Mexican blind cavefish *A. mexicanus* from Pachón, and Chica and its surface counterpart fish. Cave forms of this species recently migrated from surface to cave. Molecular phylogenetic analysis confirms their recent origin, of several tens of thousands of years ago [30]. Research revealed a high-amplitude rhythm in *per1* expression, with a peak in the late night at zeitgeber time (ZT) 21 in surface *A. mexicanus* under a 12/12-h LD cycle in the laboratory. The *per1* expression rhythm was lower in amplitude, and its peak expression occurred 6 h later in both cave populations under the same conditions. These rhythms in *per1* expression continue as the animals free-run into constant darkness [7]. At the same time, samples from wild cave environments (Chica Cave 10 km south of Ciudad Valles in the Mexican state of San Luis Potosí) showed no significant oscillation in the levels of *per1* expression in the absence of entrainment to a light–dark cycle. In addition, the expression levels of *per1* are significantly lower in cave animals under natural conditions than those measured in surface fish or equivalent cave strains within the lab [7]. Thus, *Astyanax* cavefish from this cave do not show circadian molecular oscillations in darkness but retain the ability to detect light and generate molecular oscillations under a light–dark cycle.

The pattern is different for light-induced genes, including *cry1a* and *per2*, which are known to be critical for light resetting of the circadian pacemaker [31,32]. A study in embryos revealed that the light induction of these genes is developmentally delayed in cave populations compared to surface fish [33]. The rhythmic expression of *cry1a* and *per2* in adult cavefish persists in total darkness, following entrainment to a light–dark cycle. However, the basal expression of both *cry1a* and *per2* in the dark was significantly raised in *A. mexicanus* cavefish compared with surface individuals. Thus, *cry1a* and *per2* are present in cavefish at near-maximal levels in the light stimuli absence. The lack of rhythmicity and significantly raised expression of *per2* were found in samples from wild cave environments [7]. Beale et al. suggest that the core clock mechanism is tonically repressed or damped in the cave, probably as a consequence of the overactivation of the light input pathway [7].

The raised basal levels of *per2* revealed in *A. mexicanus* cavefish have an adaptive advantage. It is known that genes encoding DNA repair proteins have been shown in surface fish to be transcriptionally activated by light [34,35]. As the *per2* expression, the basal levels of two DNA repair genes (*CPD phr* and *ddb2*) induced by light were significantly raised in *A. mexicanus* cavefish maintained in darkness compared to the surface fish. Moreover, cavefish show significantly lower DNA damage and, therefore, higher DNA repair activity in comparison with surface form after UV exposure [7]. The authors suggest that cavefish in their natural habitats would reduce deleterious mutational events and increase DNA repair activity by tonically activating light-dependent signaling pathways. In turn, it can highly repress levels of clock function in arrhythmic environments [7].

The expression of circadian genes was also studied in an ancient Somalian cavefish *P. andruzzii*, which diverged from surface-dwelling ancestors more than a million years ago [28,36]. Despite Mexican blind cavefish, *P. andruzzii* showed no circadian rhythms in the expression of circadian genes (*Clk1a, Clk2, Per1, Per2, Cry1a, Cry5*) under a 12/12-h LD cycle in the laboratory. At the same time, Somalian cavefish demonstrated robust circadian rhythms of both *Clk1a* and *Per1* genes expression in response to a periodic feed schedule as an alternative environmental zeitgeber [18].

### 2.3. Microevolutional Aspects of Circadian Clock Reduction in Cavefish

The molecular circadian clock has been studied in detail in *A. mexicanus*. Microevolutionary processes can be viewed by comparing isolated populations of this species from different caves.

Mack with co-authors [8] performed RNAseq with total RNA from whole animals in three cave populations (Molino, Pachón, and Tinaja) and one surface population (Río Choy) of Mexican tetra to identify changes in rhythmic expression between cave and surface populations. Fish were maintained in a light–dark cycle (14:10) to synchronize behavioral and molecular clocks and then transferred into constant darkness 24 h before the start of the sampling period.

They found that the surface population had the greatest number of rhythmic transcripts (539), followed by Tinaja (327), Pachón (88), and Molino (83), respectively. Only 19 genes (including key genes of the circadian clock as *per1a*, *per1b*, *cipca*, *ciarta*, *dbpb*) showed significant cycling across all cave and surface populations, i.e., some components of the core clock remain functional across cavefish populations. The authors show that the loss of rhythmicity has evolved repeatedly among independent origins of the cave phenotype. Losses of rhythmic expression were often population specific. About 22% of genes found to be rhythmic in the surface population were arrhythmic in all three cave populations, and 77% were arrhythmic in at least one cave population. On the contrary, no genes that were rhythmic across all caves were arrhythmic in the surface population [8]. The authors also revealed a relaxation of negative selection in the protein-coding sequences of clock genes (*per1b, cry4, arntl2, cry1ab*) in populations from Tinaja and Pachón caves but not from Molino cave.

Moreover, studied cave populations of A. mexicanus differed in the amplitude of rhythmic transcription of key circadian genes. However, the populations from three caves show a unidirectional shift in the phase of rhythmic transcription compared to the surface population: a delay of peak expression on 0.48–2.03 h.

As with zebrafish [37], surface and cave Mexican tetra have decentralized circadian systems. Autonomous oscillations in core clock gene expression were revealed in the brain and liver. In addition, temporal expression patterns for clock gene expression in different tissues varied between cave populations. Melatonin rhythms also differed between *A. mexicanus* from Molino, Pachón, and Tinaja caves [8].

## 3. Discussion

The circadian rhythms provide an organism fitness for the daily changing environment. At the same time, physiological advantages, such as the temporary separation of different biological processes, which makes them more efficient, are often attributed to these rhythms regardless of external signals [38]. If this last aspect is true, then evolution should have retained the rhythm in cavefish in their natural habitats [39]. However, the data for the last decades incline us to abandon this assumption. Researchers mainly revealed the rhythms of circadian gene expression in different species of cavefish in laboratory conditions under various light–dark regimes and the absence of rhythms in complete darkness. Evidence from behavioral studies is also not so unambiguous. Detection of locomotor activity rhythms in complete darkness may be a consequence of the used method’s features (see notes in Table 1).

As we wrote earlier, eyes are generally reduced in troglobites [40]. Other sensory analyzers are sharpened, helping to navigate and find food in complete darkness. Thus, sensitivity to vibrations increases in cavefish, e.g., *A. mexicanus* has specific vibration attraction behavior or the movement of cavefish toward the source of water disturbance [41] that is useful in total darkness and food scarcity [42]. Cavefish have better olfactory capacities compared to surface fish [43]. Therefore, troglobites have no close connection between the visual analyzer and the internal circadian oscillator as the fish live in a day–night rhythmic environment. It is also known that various external zeitgebers with a period close to 24 h can entrain circadian rhythms due to connections between the endogenous circadian clock and associative memory through time–place learning processes [44]. A dominant link between the visual analyzer and the circadian clock is at different stages of evolutionary destruction in species and populations of cavefish. It is possible that the emergence of new connections may occur faster in troglobites. Therefore, cavefish would facilitate the study of links between neurophysiology and circadian molecular biology. Moreover, cavefish can be utilized in studies of nonphotic zeitgebers, such as slow magnetic fluctuations, acoustic and vibrational signals, and minor temperature changes.

Aging is another area of research where cavefish are in demand. It is thought that animals from arrhythmic environments have a longer lifespan [45,46,47]. Data from cavefish evidence this suggestion. Poulson [48] showed increased longevity in populations of troglobitic amblyopsid fishes (*Typhlichthys subterraneus*, *Amblyopsis spelaea*, and *A. rosae*) compared to two surface species (*Chologaster cornuta* and *Forbesichthys agassizi*). The estimated longevity of other cavefish exceeds that of related surface species: 20–25 years in *Ancistrus cryptophthalmus* [49], up to 15 years in *Caecobarbus geertsii* and *Pimelodella kronei* [50,51,52], and more than 10 years in *Ituglanis passensis* [53]. Some researchers believe that circadian rhythms are closely related to aging [54,55]. We describe some specificities of internal circadian oscillators in cavefish in the previous section. Molecular clocks in other animals from arrhythmic environments also have their peculiarities [56]. If these inconsistencies in trilobite’s circadian oscillators are related to the increased lifespan, then cavefish can be very useful in investigating the relationship between circadian rhythms and aging.

The comparison of the expression of circadian genes in *A. mexicanus* and *P. andruzzii* shows that the ancient origin of the cave form is accompanied by the lacking of a functional light input pathway [7,18]. However, the rhythmic expression of circadian genes is not entirely lost in *P. andruzzii* and may be carried away by other zeitgebers [18]. The findings by Mack et al. [8] reveal that the molecular circadian clock has been independently disrupted in the unique origins of several cavefish populations within an *A. mexicanus* species. Most likely, the functioning of circadian oscillators in other cavefish can also differ due to other evolutionarily determined irregularities in the functioning of molecular circadian oscillators.

Unique disruptions to circadian rhythms across different origins of cavefish provide a powerful platform for studying the relationship between naturally occurring clock mutations and circadian biology. Moreover, in evolutionary history, many cave species originated due to the movement of terrestrial species to cave environments [3]. Therefore, for most cavefish, there are related species on the surface. Moreover, one species can be in two forms: surface fish and cavefish. For example, the Mexican tetra, one of the most demanded cave model species, has a surface-dwelling and a cave-dwelling form [25]. It allows for carrying out all the above studies in an evolutionary-comparative manner.

However, these cave-specified differences between populations are particularly difficult for behavior and physiology research. Suppliers of blind cavefish for experimentation do not always state their specific origin. The expression of circadian genes and the manifestation of rhythms at higher levels of the biological organization can differ significantly in populations originating from different caves due to microevolutionary processes. Thus, results of chronobiological experiments carried out with representatives of the same species from various cave populations can differ significantly. It can lead to the incomparability of data from different laboratories.

## 4. Conclusions

Cavefish are promising objects for chronobiological research. They could be utilized for studying the effects of nonphotic external circadian zeitgebers and the relationship between circadian rhythms and aging. Parallel evolutionary transformations in local closed cave populations have led to the emergence of unique modifications of circadian rhythms. It allows for comparative evolutionary studies of changes in behavioral, physiological, and molecular rhythms between different cave populations within a species. At the same time, a divergence of the circadian system between cavefish populations requires the of strain origin to be indicated in publications.

## Figures and Tables

**Table 1:** Rhythms of locomotor activity in cavefish.

Species	Degree of Troglomorphism *	Light Mode or Feeding Regime **	Periods of Rhythms, h			Arrhythmic Individuals, %	References
			Ultradian	Circadian	Infradian		
*Stygichthys typhlops*	++++	DD	-	-	-	100	[9] ^a^
*Aspidoras mephisto*	+	DD	3.8–14	24.3–25.2	-	-	[9] ^a^
*Rhamdia enfurnada*	++	DD	6.4–18.9	21.7–27.0	-	14.3	[9] ^a^
*Trichomycterus* *itacarambiensis*	++	DD	2.4–19.7	21.4–26.1	28.5–128	-	[9,10] ^a,b^
*Pimelodella spelaea*	+	DD	7.9–18.4	20.25–25.9	-	9.1	[9] ^a^
*Pimelodella kronei*	++	DD	4.6–19.7	22–27.5	28.4–128	-	[9,11] ^a,b^
*Rhamdiopsis* sp. (from Salitre Cave)	+	DD	7.7–16.5	20.66–26.7	28.25	22.2	[9] ^a^
*Rhamdiopsis* sp. (from Toca do Gonçalo cave)	++++	DD	1.1–14.5	-	-	40	[12] ^b^
		LD (12:12)	8–14	24	42	-	
		LD → DD	2.8–5.3	24	-	25	
*Rhamdiopsis krugi*	++++	DD	1–13.5	23.3–24	28.4–128	2	[13] ^c^
		LD (12:12)	1.8–12	23.58–24.25	-	-	
		LD → DD	1.1–10.9	23.4–24	-	-	
*Indoreonectes (Nemacheilus)* *evezardi*	+	LD (12:12)	12	24–26.18	-	-	[14,15] ^d^
		LD → DD	8.29–12.32	21.71–25.33	28.5	0–42.9	
		SF	-	24	-	-	
*Astyanax mexicanus*	++	LD (12:12)	-	23.95–25.6	-	-	[7,16] ^e,f^
		LD (9:9)	18	-	-	-	
		LD (3:7)	10	-	-	-	
		LD → DD	-	- ***	-	-	
*Schistura jaruthanini*	+	DD	-	23.19	-	-	[17] ^g^
*S. spiesi*	++	DD	-	24.06	-	-	[17] ^g^
*S. oedipus*	++	DD	-	-	38.5	-	[17] ^g^
*Nemacheilus* *troglocataractus*	+++	DD	-	24.92	-	-	[17] ^g^
*Phreatichthys* *andruzzii*	++++	LD (12:12)	-	-	-	100	[18] ^h^
		SF	-	24	-	-	

* The degree of troglomorphism [19]: + (low degree) eyes and pigmentation only slightly, but significantly, reduced compared to epigean congeners; ++ (moderate degree) high intra-population variation, from the epigean state to its complete absence; +++ (high degree) eyes and pigmentation conspicuously reduced, but still noticeable in at least part of the population; ++++ (very high degree) population homogeneously anophthalmic and depigmented, frequently with other morphological, physiological, and/or behavioral troglomorphisms. ** DD, constant darkness; LD, light–dark cycle; SF, scheduled feeding. *** There was no significant rhythm *A. mexicanus* in DD, but some exceptions were observed. In the DD followed an LD of 12:12 h, the activity near the surface exhibited a free-running rhythm with a period of 22.2 h, while the bottom activity was arrhythmic. DD was followed by an LD of 8:8 h, a free-running rhythm with a period of 22.5 h swung in the surface activity in half the cases [16]. a—performed in wild fish maintained in DD before the experiments, the time series were analyzed individually using the Lomb–Scargle periodogram; b—performed in wild fish maintained in the laboratory in DD before the experiments, the time series were analyzed individually using the fast Fourier transform algorithm; c—performed in wild fish maintained in DD before the experiments, the time series were analyzed individually using both the Lomb–Scargle periodogram and the fast Fourier transform algorithm; d—performed in wild fish maintained in DD before the experiments, cosinor rhythmometry and power spectrum analysis were used; e—performed in fish from a laboratory strain maintained in standard LD laboratory conditions, the time series were analyzed with autocorrelation and spectral resampling; f—performed in fish from a laboratory strain maintained in standard LD laboratory conditions; g—performed in wild-caught fish maintained in a laboratory setting on a 12:12 LD for no less than six months before the experiments, the time series were analyzed using the Lomb–Scargle periodogram; h—performed in fish from a laboratory strain maintained in the laboratory under DD, the time series were analyzed using χ2 periodogram.
